# Spectrum of liver diseases in patients referred for Fibroscan: A single center experience in the Middle East

**DOI:** 10.1016/j.amsu.2020.07.040

**Published:** 2020-07-25

**Authors:** Bisher Sawaf, Adel Hajj Ali, Rola F. Jaafar, Mariam Kanso, Deborah Mukherji, Mohamad J. Khalife, Walid Faraj

**Affiliations:** a Department of Surgery, Faculty of Medicine, American University of Beirut Medical Center Beirut, Lebanon; b Department of Internal Medicine, Faculty of Medicine, American University of Beirut Medical Center Beirut, Lebanon; c Liver Transplantation and Hepatopancreaticobiliary Surgery, Department of General Surgery, American University of Beirut Medical Center Beirut, Lebanon

**Keywords:** Fibroscan, Liver disease, Etiology, Spectrum, Lebanese population

## Abstract

**Background:**

Liver diseases is a worldwide etiology causing high morbidity and mortality. Fibroscan is a quick, painless examination performed in clinic or at the patient's bedside. It is used to evaluate liver status for patients with suspected liver disease prognosis. This study aims at describing the spectrum of liver diseases among patients performing Fibroscan at a tertiary care center in Lebanon.

**Methods:**

This is a retrospective data collection study on patients who underwent Fibroscan at the American University of Beirut hepatobiliary unit between 2015 and 2018. Medical charts of all patients were reviewed. Data were collected and analyzed using SPSS 25 software.

**Results:**

A total of 620 patients presented to the hepatobiliary unit for Fibroscan, of which 419 (67.5%) were males. The mean age was 47.8 ± 13.4 (range 18–84). 362(58.3%) had NAFLD, 89 (14.3%) had Hepatitis-B, 69 (11.1%) had Hepatitis-C, 48 (7.7%) had ALD, 20 (3.3%) had DILI, and 13 (2.9%) had autoimmune hepatitis. 190 (30.6%) were overweight (BMI over 25), 128 (20.6%) had diabetes. Liver stiffness corresponding to the diagnosis of F4 liver fibrosis stage on Fibroscan was mostly reported in 6 (46.5%) autoimmune hepatitis, 101 (27.9%) NAFLD, and 18 (26.1%) HCV patients. 141 (45.5%) patients who had one or more metabolic risk factors suffered from severe stage steatosis compared with 78 (28.9%) who had not any risk factors with *P*-value 0.04.

**Conclusions:**

Based on our sample**,** NAFLD is emerging as a predominant etiology of CLD, followed by, HBV, and HCV. This is the first study that reports CLD status in Lebanon, further studies that describe the prevalence and incidence of the disease at a larger scale are needed.

## 1 Introduction

Chronic liver disease (CLD) is a global public health problem associated with significant morbidity and mortality [1,2]. Since prognosis and management depend on the extent of liver fibrosis and progression to cirrhosis, accurate assessment of liver fibrosis is essential. Early detection of liver injury and identification of the related etiology can potentially lead to an intervention that may halt or potentially revert the process of fibrosis. This can be achieved for example by the administration of antiviral drugs for viral hepatitis, or initiation of crucial life-style changes in patients with significant liver steatosis [3]. The gold standard for staging and grading of liver fibrosis remains liver biopsy and histologic examination; however, it is an invasive procedure with potential serious complications what limits the use of liver biopsy as a procedure for screening or routine follow-up. Therefore, accurate non-invasive diagnostic tools for liver diseases are currently emerging [4]. Vibration Controlled Transient Elastography Transient elastography (VCTE) by Fibroscan (EchoSens, Paris, France) is a validated, accurate, non-invasive method for liver fibrosis staging [4]. It is a quick, painless and performed in clinic or at the patient's bedside, and is currently used to evaluate liver status for patients with liver problems such as non-alcoholic fatty liver disease (NAFLD), alcoholic liver disease (ALD), chronic hepatitis infections, cholestatic diseases, and autoimmune hepatitis [5]. Recent guidelines from European Association for the Study of the Liver (EASL) and American Association for the Study of Liver Diseases (AASLD) recommended the use of Fibroscan as clinical diagnostic tool for patients with viral hepatitis infection; with a >90% negative predictive value for ruling out cirrhosis [6]. It is also a clinical tool for identifying advanced fibrosis in patients with metabolic syndrome [7,8]. NAFLD and non-alcoholic steatohepatitis (NASH) are currently the most common etiologies for CLD in developed countries [[9], [10], [11]]; whereas, viral hepatitis is mostly observed in low- and middle-income developing countries [12]. Few studies have investigated the most prevalent causal factors, diagnosis and management of CLD in the Middle East. This is the first study in Lebanon that describes the spectrum of liver disease among Lebanese population and identifies etiologies and characteristics of patients presenting with liver problems.

## 2 Materials and methods

The study population includes all 620 patients who underwent the Fibroscan test to assess their liver status at the Liver Transplant and Hepaticopancreaticobiliry Unit at the American University of Beirut Medical Center (AUBMC) Beirut, Lebanon, between January 1, 2016 and August 31, 2018. Ethics approval was obtained from the American University of Beirut Institutional Review Board (IRB). Patients above 18 years old diagnosed with CLD, assessed for liver fibrosis by Fibroscan, were evaluated for etiology by standard clinical and laboratory criteria and grouped as ALD, NAFLD, viral liver disease, drug-induced liver injury (DILI) and others. Data collected in this retrospective study included age, sex, BMI, waist circumference comorbidities, glucose level, hemoglobin A1c (HbA1c), liver, kidney, thyroid laboratory tests in addition to the patients’ past medical history (coronary artery disease, congestive heart failure, hypertension, diabetes, chronic kidney disease and malignancies. Fibrosis and steatosis results and stages were recorded as reported by Fibroscan output.

The clinical findings were used in association with laboratory studies to calculate Child-Pugh Score. The Child-Pugh score was calculated by adding the score of five factors (ascites, bilirubin, albumin, prothrombin time and encephalopathy) and ranged from 5 to 15. Child Pugh score between 5 and 6 indicated class A; 7 to 9 indicated class B and score of 10 or above indicated class C.

### 2.1 Statistical analysis

Data were analyzed using statistical package for the social sciences (SPSS) version 25.0 software (IBM Corporation Armonk, New York, USA). Quantitative variables were summarized using descriptive statistics (number of observations, percentages, mean, standard deviation [SD]). Categorical and discrete variables are presented in percentages. Tests were done at 2-sided 5% level of significance. The work has been reported in line with the STROCCS criteria [13].

This study was registered at the Lebanese National registry portal accessible at https://lbctr.moph.gov.lb/LBCTR/Trials/Details/4524.

## 3 Results

### 3.1 Demographics and patient characteristics

620 patients were enrolled in this study. The mean age of the population was 48.7 ± 13.2 years, with 419 (67.5%) males and 201 (32.5%) females. The majority 360 (58.1%) of patients were graduate or post-graduates (Table 1).

**Table 1:** Baseline demographic characteristics.

Age (yrs.), Mean (SD)		48.7 ± 13.2
Gender	Male n (%)	419 (67.5%)
	Female n (%)	201 (32.5%)
Smoking or water pipe smoking n (%)		284 (45.8%)
BMI (Kg/m2), Mean (SD)		26.21 ± 4.3
Waist circumference (cm)		91.02 ± 10.8
Socio-economic status, n (%)		
Education	Graduate or postgraduate	360 (58.6%)
	post high school diploma High school certificate	149 (24.0%)
	Middle school or Primary school Certificate	111 (17.9%)

**Fig. 1:**
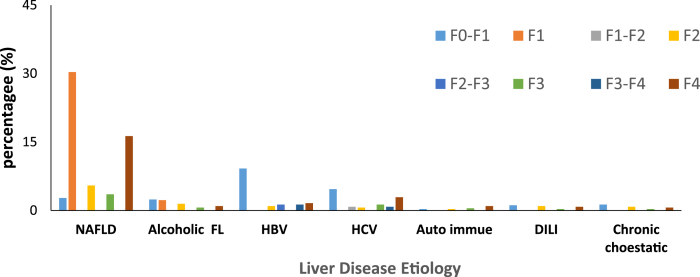
Distribution of liver diseases based on etiology and fibrosis score.

Of the 620 patients, 362 (58%) presented with NAFLD, 89 (14.4%) with HBV, 69 (11.1%) with HCV, 48 (7.4%) with ALD, 20 (3.2%) patients had DILI, 19 (3.1%) had chronic cholestatic disease and 13 (2.01%) had autoimmune hepatitis (Fig. 1).

The duration of CLD was 1.3 ± 1.2 years. Of the 620, 385 (62.1%) had associated comorbidities. 262 (42.3%) had history of alcohol consumption, obesity in 190 (30.7%) patients, diabetes mellitus Type-2 (T2DM) in 128 (20.6%), hypertension in 121 (19.5%), dyslipidemia in 90 (14.5%). The predominant presenting signs and symptoms were abdominal pain 224 (36.1%), fatigue 180 (29.0%), nausea 121 (19.5%) (Table 2) (see Table 3).

**Table 2:** Co-morbidities, Signs, symptoms, physician referral reported.

Condition	N = 620
Subjects with any comorbidity	385 (62.1%)
Details of related comorbidities	
Type 2 Diabetes Mellitus (T2DM)	128 (20.6%)
Hypertension (HTN)	121 (19.5%)
Dyslipidemia	90 (14.5%)
Alcohol Intake	262 (42.2%)
BMI > 25	190 (30.6%)
Coronary Artery Disease (CAD)	29 (4.7%)
Hypothyroidism	8 (1.3%)
Chronic Kidney disease	7 (1.1%)
Hyperchlorhydria	18 (2.9%)
Main signs and symptoms	
Abdominal pain	224 (36.1%)
Fatigue	180 (29.0%)
Appetite loss	121 (19.5%)
Specialty referral to Fibroscan	
Gastroenterology	255 (41.1%)
General Surgery	236 (38.1%)
Others	129 (20.8%)

### 3.2 Hepatic fibrosis scores among patients

The median liver stiffness was maximum observed in patients with autoimmune hepatitis, hepatitis, followed by DILI, HCV, HBV, NAFLD, chronic cholestatic disease ALD (Fig. 1). Liver stiffness corresponding towards the diagnosis of F4 liver fibrosis stage was 6 (46.5%) in autoimmune patients, 101 (27.9%) in NAFLD patients, 18 (26.1%) in HCV patients, 5 (25.0%) in ALD patients, 5 (21.0%) in patients with chronic cholestatic disease, and 10 (11.2%) in HBV patients, as reported by Fibroscan. Out of 362 patients with NAFLD, liver stiffness corresponding towards the diagnosis of F0–F2 liver disease stage was found in 205 (56.6%) patients, followed by F4 101 (27.9%), F2 34 (9.3%), and F3 22 (6.1%). Out of 48 patients with ALD, liver stiffness values corresponding to F0–F2 liver disease stage was reported in 29 (60.6%) patients, followed 9 (18.8%) in F2, then 6 (12.3%) in F4, and 4 (8.3%) in F3 patients. 54 (60.7%) of HBV patients had liver stiffness corresponding towards the diagnosis of F0–F2 liver disease stage, followed by F4 10 (11.2%), then 9 (10.1%) F3–F4, 9 (10.1%) F2–F3, and 7 (7.8%) F2. 29 (42.0%) of HCV patients (total = 69) had liver stiffness corresponding towards the diagnosis of F1 liver disease stage, followed by 18 (26.1%) F4, 8 (11.5%) F3, 5 (7.5%) F1–F2, 5 (7.5%) F3–F4, and 4 (5.8%) F2. 7 (35%) of DILI patients (n = 20), had liver stiffness corresponding towards the diagnosis of F0–F1, followed by 6 (30%) F2, 5 (25%) F4, and 2 (10%) F3. 6 (45.0%) of autoimmune hepatitis patients, had liver stiffness corresponding towards the diagnosis of F4, 3 (23%) F2, 2 (15.3%) F3, and 2 (15.3%) had F1.

### 3.3 Child-Pugh's scoring of patients

Out of the total of 167 patients whose Child-Pugh's scoring was done, the proportion of patients presented with Grade A 139 (22.8%) and Grade B 25 (4%), only 3 (0.5%) patient was presented with Grade C (Table 4).

**Table 3:** Summary of severity and staging of liver disease.

Category	Mean ± SD
VCTE results median stiffness (kPa)	25.9 ± 21.6
IQR (kPa)	4.0 ± 3.9
IQR/Med (%)	14.6 ± 9.7
Valid measurement, mean (SD)	10.8 (6.1)
Liver fibrosis/liver disease stage, n (%)	
<7kpa	336 (54.2%)
>7kpa	284 (54.8%)

**Table 4:** Summary of Child-Pugh score.

Variables		
Total Bilirubin (mg/dL)		1.4 ± 0.75
Serum albumin		1.5 ± 0.59
Prothrombin time, prolongation (s) INR		1.3 ± 0.53
Ascites		1.3 ± 0.38
Hepatic encephalopathy		1.1 ± 0.11
Total Score, n (%)	Grade A (5–6)	139 (22.4%)
	Grade B (7–9)	25 (4.03%)
	Grade C (10–15)	3 (0.48%)

### 3.4 Steatosis in liver disease patients

Comparing patients with one or more risk factors to those who didn't, patients with steatosis was significantly higher in NAFLD group compared to others, regardless of the cutoff values of CAP used for steatosis grading. Of the 620 patients, 579 (93.3%) had reported steatosis, 310 (50.0%) patients had one or multiple metabolic risk factors such as hypertension, T2DM, dyslipidemia, and overweight. Among these subjects, mild and moderate steatosis was minimal 38 (12.0%) patients in the risk group. The presence of severe steatosis (stage 3) was 141 (45.5%) in NAFLD patients. (Table 5).

**Table 5:** Prevalence of steatosis in patients with NAFLD and with other liver etiology.

	NAFLD n = 310	others n = 269	*P*-value
*No steatosis*	*131 (42.2%)*	*112 (41.6%)*	*0.04*
*Steatosis*	*179 (57.7%)*	*157 (58.4%)*	*0.03*
*S1*	*16 (5.2%)*	*45 (16.7%)*	*0.00*
*S2*	*22 (7.0%)*	*34 (12.6%)*	*0.00*
*S3*	*141 (45.5%)*	*78 (28.9%)*	*0.04*

## 4 Discussion

In our population, a higher percentage of patients with suspected CLD were males, similar to the results reported by Indian studies they found that 67.9% of their patients were males [14]. Moreover, the results of Mukherjee et al. and Jhajharia et al. had reported 78.6% and 80.6% males in these studies [15,16]. These results indicate that males are more susceptible to CLD than females in general, suggesting a high risk of exposure to causative factors. Moreover, the mean age of patients was 47.8 which is consistent with the finding of Jhajharia et al. where the mean age of presentation was 45.6 [16].

NAFLD was the main etiology of CLD in Lebanon, similar to the worldwide trend of high NAFLD prevalence. Other studies showed that more than 80% of CLD were of viral etiology and 11% alcohol [17].

Another prospective study from Greece in CLD population demonstrated that 44% of the subjects had chronic HBV, 40% had HCV, alcoholic (8.7%) [18]. These differences in these studies could be attributed to the observed differences in CLD prevalence across countries and differences in contributing risk factors, which may vary significantly based on the geographical regions.

This etiological spectrum of CLD indicated in this study shows the epidemiological transition within the Middle East in general and Lebanon in specific. Further, a higher proportion of NAFLD reported in our study could be attributed to the expected reason. It could be explained by the fact that AUBMC is a tertiary care center with the majority of its patients belonging to well educated, professional upper middle class in society, making them less susceptible to alcohol abuse or addiction [19].

The correlation between NAFLD and components of metabolic syndrome has been strongly established. Hence, the increase in metabolic risk factors including T2DM, HTN, obesity, and dyslipidemia in Lebanon population could also be the contributing factor for this relatively high NAFLD in the region and in Lebanon [20]. By considering the components of the metabolic syndrome such as hypertension, T2DM dyslipidemia and increased body mass index, NAFLD may be considered as hepatic manifestation of this syndrome. This study also reported an increase in of associated comorbidities, such as T2DM, hypertension, hypothyroidism, dyslipidemia, and obesity in the risk group.

The mode of presentation of patients with CLD was an important consideration taken in our study. Abdominal pain, fatigue, and decreased appetite were the major sign and symptoms associated with CLD patients in our study. These symptoms are markers of hepatic disease advanced. This study has contrast the result of the study of Chandra et al. who has noted ascites in 52% of patients followed by jaundice in 40% and GI bleeding in 30% as the sign and symptoms associated with CLD [15].

Liver stiffness corresponding towards the diagnosis of F4 liver fibrosis stage was reported in 46.15% auto immune hepatitis, 27.9% NAFLD, 26.08% HCV and 25% DILI and chronic cholestatic disease, 12.3% of ALD, 11.2% of HBV patients [[21], [22]]. However, Majority of 64.04% HBV, 42.02% HCV, 40% chronic cholestatic disease, 29.4% ALD patients reported in F0–F1 stage. This signifies that majority of patients detect in early stages.

Child-Pugh score and class, a marker of liver damage, was reported in this study. Pal et al. study found that 51% of patients belonged to Child-Pugh class B, followed by class C in 35% and only 14% in class A. Chandra et al. study also found that Child-Pugh class B and C together constituted more than 81% of their patients, which is consisted of advanced liver disease. In this study, out of 167 patients whose Child Pugh's scoring was reported (around 27% of whole patients), presented with Grade A 139 (22.7%) and Grade B 25 (4.0%), only 3 (0.4%) patient was presented with Grade C (Table 4).

To conclude, this study indicates that NAFLD is emerging as an important etiology of CLD in Lebanese real-world setting, followed by HBV, HCV, ALD and others. Significant regional differences. CLD in Lebanon has a male preponderance, affecting mostly people of the middle age group. Considerable percentage of the patients had early to advance fibrosis stages, based on VCTE assessment. This study emphasizes the need for appropriate risk evaluation in more detail and more assessment of the severity of the liver disease. However, as this was a cross-sectional study, the cause and effect of relationship could not be determined in this study. Hence further multicenter prospective studies are warranted in larger and more representative samples to improve the generalizability of the findings in Lebanon and Middle East in general.

## Ethical approval

Ethical approval of the study was approved by the institutional review board (IRB) at the American University of Beirut Medical Center (AUBMC) in Beirut, Lebanon. It was rated as a human subject study. All procedures contributing to this work comply with the ethical standards of the relevant national and institutional committees on human experimentation and with the Helsinki Declaration of 1975, as revised in 2008.

## Sources of funding

None.

## Authors’ contributions

BS was responsible for study analysis and write up; RJ conceptualized the study, participated in the design, wrote the study protocol, and reviewed final version of manuscript; MK participated in the design, did literature search and revision of draft. BS participated in literature search, supervised data collection and write the draft. FD, DM & WF participated in design, literature search and revision of draft. All authors read and approved the final draft.

## Trial registry number

Not required for retrospective non-identified data study.

Name of the registry: Lebanese Clinical Trials registry.

Unique Identifying number or registration ID: LBCTR2020074524.

Hyperlink to your specific registration (must be publicly accessible and will be checked): https://lbctr.moph.gov.lb/LBCTR/Trials/Details/4524.

## Guarantor

Walid Faraj

wf07@aub.edu.lb.

## Consent

Not applicable.

## Availability of data and materials

All data related to this paper's conclusion are available and stored by the authors. All data are available from the corresponding author on reasonable request.

## Provenance and peer review

Not commissioned, externally peer reviewed.

## Declaration of competing interest

None of the authors have any competing interests. The authors alone are responsible for the content and writing of the article. No conflict of interest is declared.

## Abbreviations

AASLD: American Association for the Study of Liver Diseases
ALD: Alcoholic Liver Disease
AUBMC: American University of Beirut Medical Center
BMI: Body Mass Index
CAP: Controlled attenuation parameter
CLD: Chronic liver disease
DILI: Drug Induced Liver Injury
EASL: European Association for the Study of the Liver
HBV: Hepatitis B Virus
HCV: Hepatitis C Virus
HTN: Hypertension
IRB: Institutional Review Board
LSM: liver stiffness measurements
NAFLD: Non-Alcoholic Fatty Liver Disease
NASH: Non-Alcoholic steatohepatitis
SPSS: Statistical Package for Social Sciences
T2DM: Type 2 diabetes mellitus
VCTE: Vibration Controlled Transient Elastography
